# Supplementary material for: Efflux pumps mediate changes to fundamental bacterial physiology via membrane potential
Source: mBio. 2024 Sep 9;15(10):e02370-24. doi: 10.1128/mbio.02370-24 (PMC11481890; doi:10.1128/mbio.02370-24)
Supplement: Supplemental material — Tables S1-S5; Fig. S1 and S2. [file mbio.02370-24-s0001.pdf]

SUPPLEMENTAL MATERIAL

Efflux pumps mediate changes to fundamental bacterial physiology via membrane potential

Emily E Whittle[1], Oluwatosin Orababa[1†], Alexander Osgerby[2], Pauline Siasat[1], Sarah J Element[1], Jessica MA Blair[1]* and Tim W Overton[2]*

[1] Institute of Microbiology and Infection, College of Medical and Dental Sciences and [2]School of Chemical Engineering, University of Birmingham, Edgbaston, Birmingham, B15 2TT, UK

† Present address: School of Life Sciences, The University of Warwick, Coventry, CV4 7AL

* To whom correspondence should be addressed:

Jessica MA Blair: j.m.a.blair@bham.ac.uk

Tim W Overton: t.w.overton@bham.ac.uk

Supplemental Tables S1-S5

Supplemental Figures S1-S2

Supplemental references

Supplemental Table S1. Genes in group one - Genes that are less downregulated at 3hr in Δ*acrB* than wild type – "late repression" pattern

Log_2 fold change				Δ*acrB*-wt Δ$\log_2$								
**wt 1vs3**	**wt 3vs5**	**Δ*acrB* 1vs3**	**Δ*acrB* 3vs5**	**1vs3**	**3vs5**	**ID**	**Gene name**	**Operon structure**	**STM**	**Product**	**Comments**	**Regulation in *E. coli***
-4.29	0.00 †	-0.53	-3.94	3.77	-3.94	RS12415	tRNA0043	tRNA0040-43		tRNA-Lys (ttt)	tRNA & related	
-3.53	0.82	0.22	-2.95	3.75	-3.77	RS13050	*suhB*	*suhB*	STM2546	Nus factor; inositol-1-monophosphatase	Translation	
-4.08	-0.65 †	-0.36	-4.08 †	3.73	-3.43	RS19775	*yidD*	*rpmHA-yidDC*	STM3841	membrane protein insertion efficiency factor	Envelope – IM protein insertion	
-3.96	0.22 †	-0.36	-3.26	3.60	-3.47	RS03415	tRNA0010	tRNA0013-07		tRNA-Gln (ttg)	tRNA & related	
-4.31	0.04 †	-0.93	-3.35	3.37	-3.39	RS03420	tRNA0011	tRNA0013-07		tRNA-Gln (ttg)	tRNA & related	
-3.86	-0.51 †	-0.54	-3.07	3.32	-2.56	RS10300	tRNA0033	tRNA0033		tRNA-Asn (gtt)	tRNA & related	
-3.75	0.27 †	-0.55	-2.96	3.20	-3.23	RS03430	tRNA0013	tRNA0013-07		tRNA-Met (cat)	tRNA & related	
-3.58	0.20 †	-0.43	-3.28	3.15	-3.47	RS20225	tRNA0067	tRNA0067		tRNA-His (gtg)	tRNA & related	
-3.44	-0.13 †	-0.43	-3.25	3.01	-3.11	RS12410	tRNA0042	tRNA0040-43		tRNA-Val (tac)	tRNA & related	
-2.45	0.87	0.40	-2.22	2.85	-3.09	RS22345	tRNA0080	tRNA0080-82		tRNA-Gly (gcc)	tRNA & related	
-2.18	0.51 †	0.67	-2.19	2.84	-2.70	RS04610	tRNA0019	tRNA0019		tRNA-Ser (gga)	tRNA & related	
-3.29	-0.17 †	-0.47	-3.39	2.82	-3.22	RS14630	tRNA0050	tRNA0050-46		tRNA-Ser (gct)	tRNA & related	
-2.37	0.76	0.41	-1.90	2.78	-2.66	RS22355	tRNA0082	tRNA0080-82		tRNA-Gly (gcc)	tRNA & related	
-3.33	0.01 †	-0.55	-2.72 †	2.78	-2.73	RS16590	*rpsU*	*rpsU*	STM3029	30S ribosomal protein S21	Ribosome	ppGpp-DksA, LexA repressed
-3.37	0.25 †	-0.72	-2.74	2.64	-3.00	RS01270	tRNA0002	tRNA0002		tRNA-Ala (tgc)	tRNA & related	
-3.08	0.34 †	-0.44	-2.78	2.64	-3.12	RS20220	tRNA0066	tRNA0066-69		tRNA-Arg (ccg)	tRNA & related	
-3.13	0.83	-0.50	-1.71	2.63	-2.54	RS15255	*queD*	*queD*	STM2949	6-carboxytetrahydropterin synthase	tRNA & related	Nac repressed
-3.29	0.16 †	-0.68	-2.17	2.61	-2.33	RS20500	tRNA0070	tRNA0070		tRNA-Ile (gat)	tRNA & related	
-3.19	-0.48 †	-0.59	-2.74	2.60	-2.26	RS01265	tRNA0001	tRNA0001		tRNA-Ile (gat)	tRNA & related	
-3.24	0.20 †	-0.66	-2.13	2.58	-2.32	RS06145	*cspF*	*cspF*	STM1243	Cold shock-like protein	SPI-11	
-1.88	1.30	0.67	-1.72	2.55	-3.02	RS13200	*yfhL*	*yfhL*	STM2576	Putative ferrodoxin	Unknown	Nac repressed
-3.19	0.08 †	-0.68	-2.22	2.51	-2.30	RS21225	tRNA0067	tRNA0066-69		tRNA-Ile (gtg)	tRNA & related	
-3.33	0.46 †	-0.84	-2.39	2.49	-2.85	RS21230	tRNA0068	tRNA0066-69		tRNA-Ala (cag)	tRNA & related	
-2.85	1.62	-0.40	-1.17	2.45	-2.78	RS04505	*artP*	*artPJQM*	STM0891	arginine ABC transporter ATP-binding protein ArtP	AA metabolism - Arginine uptake	ArgR repressed, Lrp regulated

-3.01	-0.49 †	-0.57	-2.70	2.44	-2.21	RS04630	*infA*	*infA*	STM0953	translation initiation factor IF-1	Translation	ppGpp repressed
-3.13	-0.69	-0.70	-2.97	2.43	-2.28	RS05850	*rluC*	*rluC*	STM1187	23S rRNA pseudouridine(955/2504/2580) synthase RluC	Ribosome	ppGpp repressed
-1.60	0.00 †	0.81	-2.98	2.41	-2.98	RS16610	tRNA0055	tRNA0055		tRNA-Ile (cat)	tRNA & related	
-3.24	0.16 †	-0.86	-2.37	2.39	-2.53	RS20505	tRNA0071	tRNA0071		tRNA-Ala (tgc)	tRNA & related	
-3.21	0.75	-0.83	-1.70	2.38	-2.45	RS07340	*ydgI*	*ydgI*	STM1477	Putative arginine:ornithine antiporter	AA metabolism - Arginine import	ArgR repressed
-2.67	-0.09 †	-0.39	-2.08	2.28	-1.99	RS08775	*lolB*	*lolB-ispE*	STM1778	Lipoprotein localization protein LolB	Envelope – OM lipoprotein trafficking	
-2.83	-0.43 †	-0.58	-2.72	2.25	-2.28	RS09715	tRNA0028	tRNA0030-28		tRNA-Leu (taa)	tRNA & related	
-3.04	-0.33 †	-0.82	-2.44	2.22	-2.10	RS04235	*ldtB*	*ldtB*	STM0837	L'D-transpeptidase	Envelope – Peptidoglycan remodelling	Rob activated, CRP regulated
-2.49	-2.18	-0.29	-4.11 †	2.20	-1.93	RS27065	*yoeI*	*yoeI-plaP*		Membrane protein	Unknown	
-2.75	-0.39 †	-0.60	-2.43	2.14	-2.04	RS23375	*rsmC*	*rsmC*	STM4556	16S rRNA (guanine(1207)-N(2))-methyltransferase RsmC	Ribosome	
-2.83	0.62 †	-0.70	-1.80	2.13	-2.42	RS06595	*ydiY*	*ydiY*	STM1327	YdiY family protein, putative OMP	Unknown	Nac repressed
-2.75	-0.06 †	-0.62	-2.35	2.13	-2.29	RS09720	tRNA0029	tRNA0030-28		tRNA-Cys (gca)	tRNA & related	
-2.72	0.11 †	-0.59	-2.07	2.13	-2.18	RS09725	tRNA0030	tRNA0030-28		tRNA-Gly (gcc)	tRNA & related	
-2.61	-0.55 †	-0.55	-2.01	2.06	-1.46	RS24830	RtT RNA			RtT sRNA	Regulation	
-2.52	1.59	-0.47	-0.96	2.05	-2.54	RS02070	*queA*	*queA*	STM0404	tRNA preQ1 (34) S-adenosylmethionine ribosyltransferase-isomerase QueA	tRNA & related	
-1.11	0.31 †	0.88	-1.64	1.99	-1.95	RS10975	*trhP (yegQ)*	*trhP*	STM2136	tRNA wobble base hydroxylation protein TrhP	tRNA & related	
-2.27	-1.70	-0.29	-3.65	1.98	-1.95	RS19205	*rpmB*	*rpmB*	STM3728	50S ribosomal protein L28	Ribosome	ppGpp / DksA repressed
-2.34	-1.78	-0.39	-3.73 †	1.96	-1.95	RS19765	*rpmH*	*rpmHA-yidDC*	STM3839	50S ribosomal protein L34	Ribosome	ppGpp repressed
-2.82	-1.06	-0.89	-3.01	1.94	-1.95	RS04740	*ycaO*	*ycaO*	STM0975	30S ribosomal protein S12 methylthiotransferase accessory protein YcaO	Ribosome	
-2.81	-0.73	-0.88	-2.25	1.93	-1.52	RS11365	*yeiU (lpxT)*	*yeiRU*	STM2213	Phosphatase PAP2 family protein	Envelope - Lipid A modification	
-2.47	-0.74 †	-0.55	-2.95	1.92	-2.21	RS17070	*rplU*	*rplU-rpmA*	STM3304	50S ribosomal protein L21	Ribosome	ppGpp / DksA, Nac repressed; MlrA activated
-1.22	-0.49 †	0.69	-1.68	1.91	-1.19	RS23015	tRNA0083	tRNA0083		tRNA-Leu (caa)	tRNA & related	
-2.32	1.21 †	-0.48	-0.43 †	1.84	-1.64	RS03590	*dtpD*	*dtpD*		Putative MFS transporter, homology to *E. coli* dipeptide importer *dtpD*	Transport	
-2.12	0.03 †	-0.27	-1.79	1.84	-1.82	RS04310	*rimO*	*rimO*	STM0852	30S ribosomal protein S12 methylthiotransferase accessory protein RimO	Ribosome	
-2.41	-1.63	-0.59	-3.52 †	1.82	-1.89	RS00220	*rpsT*	*rpsT*	STM0043	30S ribosomal protein S20	Ribosome	ppGpp / DksA repressed
-2.37	-0.70	-0.60	-2.19	1.78	-1.49	RS17005	*secG*	*secG*- tRNA0057	STM3293	Preprotein translocase subunit	Envelope - Protein translocation	Nac repressed
-2.97	-0.41 †	-1.20	-2.11	1.77	-1.70	RS19950	*mioC*	*mioC*	STM3875	FMN-binding protein	Cell division	
-2.46	-0.20 †	-0.70	-2.15	1.76	-1.94	RS04765	*cmk*	*cmk*	STM0980	Cytidylate kinase	Nucleotide metabolism - Pyrimidine salvage	
-2.46	-1.17	-0.70	-2.10	1.76	-0.93	RS22135	RS22135	RS22150-22145-26870-22135	STM4312	Hypothetical protein	Unknown - Regulatory target of HilD (1)	

-2.30	0.52	-0.54	-1.38	1.75	-1.90	RS08675	*yciA*	*yciA*	STM1736	Acyl-CoA thioester hydrolase	Unknown	
-2.33	-0.45 †	-0.58	-2.53	1.75	-2.08	RS17000	tRNA0057	*secG*- tRNA0057		tRNA-Leu (gag)	tRNA & related	
-2.38	-1.88	-0.63	-3.42	1.75	-1.54	RS19200	*rpmG*	*rpmG*	STM3727	50S ribosomal protein L33	Ribosome	ppGpp / DksA repressed
-2.33	1.35	-0.60	0.17 †	1.73	-1.18	RS19380	*mgtC*	*mgtC*	STM3764	Regulator of phosphate uptake	Phosphate uptake	
-2.38	0.31 †	-0.65	-1.50	1.73	-1.81	RS00940	*gluQRS*	*dksA-gluQRS*	STM0185	tRNA glutamyl-Q(34) synthetase	tRNA & related	ppGpp / DksA repressed
-1.98	-1.13	-0.25	-2.70	1.72	-1.57	RS16385	*ygiR*	*ygiR*	STM3168	YgiQ family radical SAM protein	Unknown	
-2.19	-1.84	-0.49	-3.65	1.71	-1.81	RS10640	*plaP*	*yoeI-plaP*	0	Putrescine/proton symporter	Polyamines - Putrescine import	
-2.38	-1.91	-0.68	-2.59	1.70	-0.68	RS21335	RS21335	RS21335	STM4156	Hypothetical protein	Unknown	
-1.34	-0.72 †	0.36	-2.04	1.70	-1.32	RS08885	*prs*	*prs*	STM1780	Ribose-phosphate pyrophosphokinase	Nucleotide metabolism - Pyrimidine biosynthesis	PurR repressed
-2.10	-0.49 †	-0.44	-2.02	1.66	-1.53	RS19185	*waaA / kdtA*	*waaA-coaD*	STM3724	3-deoxy-D-manno-octulosonic acid transferase	Envelope - LPS synthesis	ArgR repressed
-2.15	-0.59 †	-0.49	-2.26	1.66	-1.68	RS12945	*trmG/rlmN*	*trmG*	STM2525	Bifunctional tRNA (adenosine(37)-C2)-methyltransferase TrmG/ribosomal RNA large subunit methyltransferase	tRNA & related	
-2.06	-0.08 †	-0.40	-2.18	1.65	-2.10	RS19040	*trmL*	*trmL*	STM3695	tRNA (uridine(34)/cytosine(34)/5-carboxymethylaminomethyluridine(34)-2'-O)-methyltransferase	tRNA & related	
-2.30	-0.90	-0.65	-2.05	1.65	-1.15	RS07240	*rsxC*	*rsxABCDGE-nth*	STM1457	Electron transport complex subunit RsxC	ROS defence	
-2.43	-0.56 †	-0.78	-1.90	1.64	-1.34	RS07250	*rsxA*	*rsxABCDGE-nth*	STM1459	Electron transport complex subunit RsxA	ROS defence	
-2.40	-0.38 †	-0.76	-1.84	1.64	-1.47	RS02460	*apt*	*apt*	STM0483	Adenine phosphoribosyltransferase	Nucleotide metabolism - Purine salvage	GlaR repressed
-2.26	-0.81	-0.63	-2.37	1.63	-1.57	RS10925	*yegD*	*yegD*	STM2125	Putative molecular chaperone	Unknown	
-2.02	-1.75	-0.40	-3.09	1.62	-1.33	RS17485	*fis*	*dusB-fis*	STM3385	DNA-binding transcriptional regulator Fis	Regulation	IHF activated, ppGpp / DksA and Fis repressed
-2.24	-0.29 †	-0.64	-1.75	1.60	-1.47	RS26790	*yqgC*	*yqgC*	STM3088	Putative cytoplasmic protein	Unknown	
-1.98	-0.90 †	-0.39	-2.14	1.59	-1.24	RS07105	*purR*	*purR*	STM1430	HTH-type transcriptional repressor PurR	Nucleotide metabolism - Purine biosynthesis	PurR, Fur repressed
-1.30	-1.11	0.28	-2.25	1.58	-1.14	RS18685	*yjhV*	*yjhV*	STM3625	Putative transporter	Phage related	
-2.26	-0.56 †	-0.69	-2.33	1.58	-1.77	RS15365	*sdaC*	*sdaCB*	STM2970	HAAAP family serine/threonine permease	AA metabolism - Serine uptake	Lrp repressed
-1.91	1.00	-0.34	-0.77	1.57	-1.77	RS13225	*rnc*	*rnc-era-recO-pdxJ-acpS*	STM2581	ribonuclease III	Ribosome	
-1.17	1.64	0.38	-0.12 †	1.56	-1.76	RS14025	*dprA*	*dprA*	STM3405	DNA-processing protein DprA	Transformation	
-2.06	0.07 †	-0.52	-1.54	1.55	-1.61	RS02175	*thiI*	*thiI*	STM0425	tRNA 4-thiouridine(8) synthase ThiI	tRNA & related	SAM repressed
-1.86	0.04 †	-0.32	-1.67	1.54	-1.71	RS16645	*rlmG*	*rlmG*	STM3220	23S rRNA (guanine(1835)-N(2))-methyltransferase RlmG	Ribosome	
-2.15	-0.82	-0.62	-1.78	1.53	-0.96	RS07235	*rsxD*	*rsxABCDGE-nth*	STM1456	Electron transport complex subunit RsxD	ROS defence	
-1.87	-1.40	-0.34	-3.02	1.52	-1.62	RS21275	tRNA0076	tRNA0076-77		tRNA-Gly (tcc)	tRNA & related	
-2.29	-2.07	-0.77	-2.25	1.52	-0.17	RS22145	RS22145	RS22150-22145-26870-22135	STM4314	GerE family regulatory protein	Unknown - Operon is regulatory target of HilD (1)	

-1.81	-1.83	-0.32	-3.06	1.50	-1.23	RS18890	*avtA*	*avtA*	STM3665	Valine - pyruvate transaminase	AA metabolism - Alanine biosynthesis	Lrp regulated
-1.84	-1.32 †	-0.37	-2.72 †	1.46	-1.41	RS03170	*pagP*	*pagP*	STM0628	Lipid IV(A) palmitoyltransferase PagP	Envelope - Lipid A biosynthesis - PhoP activated in Salmonella (2)	PhoP, SlyA activated, H-NS repressed
-2.19	-2.32	-0.72	-3.52	1.46	-1.21	RS00840	*speD*	*speED*	STM0165	Adenosylmethionine decarboxylase	Polyamines - Spermidine synthesis	
-2.11	-0.37 †	-0.66	-1.48	1.45	-1.11	RS21255	*coaA/panK*	*coaA*	STM4139	Type I pantothenate kinase	CoA synthesis	
-1.89	2.92	-0.44	-0.70 †	1.45	-3.62	RS12265	*fadL*	*fadL*	STM2391	Long-chain fatty acid transporter FadL	Fatty acid uptake	Lrp, PdhR, CRP, PhoP, ppGpp activated, RpoN, ArcA, FadR, OmpR, Rob repressed.
-1.71	-2.21	-0.27	-3.25	1.44	-1.04	RS19780	*yidC*	*rpmHA-yidDC*	STM3842	Membrane protein insertase YidC	Envelope – IM protein insertion	
-1.81	-0.35 †	-0.38	-1.69	1.42	-1.34	RS08595	*rluB*	*rluB*	STM1719	23S rRNA pseudouridine(2605) synthase RluB	Ribosome	
-1.72	-0.45 †	-0.31	-1.73	1.41	-1.28	RS08855	*sirB2*	*hemA-prfA-prmC-sirB2B1-kdsA*	STM1774	SirB family protein	Linked to SPI-1?	
-2.15	-0.94 †	-0.75	-2.63	1.41	-1.69	RS15370	*sdaB*	*sdaCB*	STM2971	L-serine ammonia-lyase	AA metabolism - Serine degradation	
-1.96	-2.10	-0.56	-3.01 †	1.40	-0.91	RS13800	*rpsP*	*rpsP*	STM2676	30S ribosomal protein S16	Ribosome	
-1.14	0.71	0.26	-0.47	1.39	-1.17	RS23350	RS23350	RS23350	STM4551	GGDEF domain-containing protein	Signalling?	
-2.60	-1.02	-1.24	-2.15	1.36	-1.12	RS04770	*rpsA*	*rpsA*	STM0981	30S ribosomal protein S1	Ribosome	ppGpp / DksA repressed
-1.62	-1.60	-0.27	-2.98	1.35	-1.37	RS17800	*rpsL*	*rpsL*	STM3448	30S ribosomal protein S12	Ribosome	ppGpp / DksA repressed
-1.90	-0.67	-0.55	-2.48	1.34	-1.81	RS00045	*satP*	*satP*	STM0009	Acetate / succinate symporter	Acetate homeostasis	
-1.86	-2.47	-0.54	-3.43	1.32	-0.96	RS22540	*rpsF*	*rpsF-priB-rpsR*	STM4391	30S ribosomal protein S6	Ribosome	ppGpp repressed
-2.10	-1.47	-0.79	-2.73	1.32	-1.25	RS16955	*rpsO*	*rpsO*	STM3283	30S ribosomal protein S15	Ribosome	ppGpp repressed
-2.04	1.86	-0.73	0.04 †	1.32	-1.82	RS12245	*sixA*	*sixA*	STM2387	Phosphohistidine phosphatase SixA	Signalling	
-2.36	1.01 †	-1.05	-1.19	1.31	-2.20	RS12950	*ndk*	*ndk*	STM2526	Nucleoside-diphosphate kinase	Nucleotide metabolism - salvage / biosynthesis	ArcA repressed
-2.00	2.18	-0.70	0.47 †	1.30	-1.70	RS08955	*cbdX*	*appCB-cbdX*	STM1794	Cytochrome bd-II oxidase subunit CbdX	Respiration	AppY, ArcA, YdeO activated
-3.41	-1.21	-2.12	-1.31	1.29	-0.10	RS14945	*spaS*	SPI-1	STM2887	SPI-1 type III secretion system export apparatus protein SpaS	SPI-1	
-1.73	-2.62	-0.45	-3.47	1.28	-0.85	RS22550	*rpsR*	*rpsF-priB-rpsR*	STM4393	30S ribosomal protein S18	Ribosome	ppGpp repressed
-1.84	-0.66 †	-0.56	-1.96	1.27	-1.29	RS02715	*lpxH*	*ppiB- lpxH*	STM0535	UDP-2,3-diacylglucosamine diphosphatase	Envelope - Lipid A synthesis	
-2.42	0.02 †	-1.15	-0.90	1.27	-0.92	RS06140	*envE*	*envE*	STM1242	Lipoprotein	SPI-11	
-2.40	-1.25	-1.14	-1.91	1.27	-0.66	RS14855	*orgB*	SPI-1	STM2868	Oxygen-regulated invasion protein	SPI-1	
-1.81	-0.69	-0.55	-1.78	1.26	-1.09	RS07245	*rsxB*	*rsxABCDGE-nth*	STM1458	Electron transport complex subunit RsxB	ROS defence	
-1.77	-0.43 †	-0.53	-1.97	1.25	-1.54	RS18495	*pitA*	*pitA*	STM3589	Inorganic phosphate transporter PitA	Phosphate uptake	FNR activated
-1.65	-1.93	-0.41	-3.14	1.24	-1.20	RS00845	*speE*	*speED*	STM0166	Polyamine aminopropyltransferase	Polyamines - Spermidine synthesis	
-1.69	-0.12 †	-0.46	-1.81	1.24	-1.70	RS27005	*yoaK*	*yoaK*		YoaK family small membrane protein	Unknown	
-1.78	-2.81	-0.56	-3.58	1.23	-0.77	RS22545	*priB*	*rpsF-priB-rpsR*	STM4392	Primosomal replication protein N	DNA replication	ppGpp repressed in coli

-2.19	0.04 †	-0.96	-1.08	1.23	-1.13	RS01120	*ispU*	*ispU-cdsA-resP-bamA*	STM0221	(2E,6E)-farnesyl-diphosphate-specific ditrans,polycis-undecaprenyl-diphosphate synthase	UPP synthesis	
-2.05	-2.33	-0.83	-1.79	1.22	0.55	RS26870	RS26870	RS22150-22145-26870-22135	STM4313	Hypothetical protein	Unknown - Regulatory target of HilD (1)	
-1.64	-0.03 †	-0.42	-0.91	1.22	-0.88	RS23295	RS23295	RS23270-95	STM4540	SIS domain-containing protein	PTS system?	
-2.14	-0.16 †	-0.92	-1.36	1.22	-1.20	RS19935	*atpI*	*atpIBEFHAGDC*	STM3872	F0F1 ATP synthase subunit I	Energy metabolism	
-1.95	-0.48 †	-0.73	-1.34	1.22	-0.86	RS08500	*rnb*	*rnb*	STM1702	Exoribonuclease II	RNA processing	
-3.31	-1.23	-2.10	-1.43	1.22	-0.20	RS14950	*spaR*	SPI-1	STM2888	SPI-1 type III secretion system export apparatus protein SpaR	SPI-1	
-1.50	1.01	-0.29	-0.39 †	1.21	-1.39	RS08250	RS08250	RS08250-55		Pseudogene	Unknown	
-1.52	-0.01 †	-0.31	-1.31	1.21	-1.30	RS07125	*rnt*	*rnt*	STM1434	Ribonuclease T	RNA processing	
-4.02	-0.03 †	-2.82	-1.46	1.20	-1.43	RS22130	RS22130	RS22130	STM4310	YjiK family protein/ putative inner membrane protein	Unknown - Regulatory target of HilD (1)	
-2.90	-1.08	-1.72	-0.96	1.18	0.12	RS14975	*spaM*	SPI-1	STM2893	SPI-1 type III secretion system protein	SPI-1	
-1.45	-0.79	-0.26	-1.65	1.18	-0.86	RS17420	*mreC*	*mreBCD*-RS17410-*rng*	STM3373	Rod shape-determining protein	Cell division / shape control	Nac, BolA repressed
-1.95	-2.47	-0.77	-3.02	1.18	-0.55	RS13795	*rimM*	*rpsP-rimM-trmD-rplS*	STM2675	Ribosome maturation factor	Ribosome	ppGpp / DksA, FNR repressed
-1.94	1.53	-0.77	0.09 †	1.17	-1.44	RS04440	*potF*	*potFGHI*	STM0877	Spermidine/putrescine ABC transporter substrate binding protein	Polyamines - Putrescine import	ArgR repressed, Lrp regulated, NtrC activated ArcA regulated
-1.81	-0.61	-0.65	-1.72	1.16	-1.10	RS09220	*proQ*	*proQ-prc*	STM1846	RNA chaperone ProQ	RNA processing	
-1.36	-2.19	-0.20	-2.95	1.16	-0.76	RS11425	*rplY*	*rplY*	STM2224	50S ribosomal protein L25	Ribosome	ppGpp / DksA repressed
-2.09	-1.21	-0.93	-1.94	1.15	-0.73	RS15975	*yqgB*	*yqgB-speA*	STM3087	Acid stress response protein	Acid stress	
-1.56	-0.39 †	-0.41	-1.64	1.15	-1.25	RS23380	*holD*	*holD-rimI-yjjG*	STM4557	DNA polymerase III subunit ψ	DNA replication	
-1.82	-0.42 †	-0.67	-1.65	1.15	-1.23	RS22410	*yjeT*	*yjeT*	STM4365	DUF2065 domain-containing protein	Unknown	
-1.68	-2.39	-0.54	-3.01	1.14	-0.62	RS17280	*rplM*	*rplM-rpsI*	STM3345	50S ribosomal protein L13	Ribosome	ppGpp / DksA repressed
-1.80	-2.40	-0.66	-3.27	1.14	-0.87	RS22555	*rplI*	*rpsF-priB-rpsR-rplI*	STM4394	50S ribosomal protein L9	Ribosome	CRP activated, ppGpp repressed
-1.57	-1.04	-0.43	-1.93	1.14	-0.90	RS05880	*plsX*	*plsX-fabHDG*	STM1192	Phosphate acyltransferase PlsX	Fatty acid metabolism?	
-1.35	-0.35 †	-0.22	-1.11	1.13	-0.77	RS19285	*recG*	*gmk-rpoZ-spoT-trmH-recG*	STM3744	ATP-dependent DNA helicase RecG	DNA repair	CreB activated, ppGpp / DksA repressed
-1.57	-0.97	-0.44	-1.75	1.13	-0.78	RS04890	*pcnB*	*pcnB-folK*	STM0184	Nicotinate phosphoribosyltransferase	Plasmid copy number	ppGpp / DksA repressed
-2.32	-1.04	-1.20	-2.06	1.12	-1.02	RS04150	*rhlE*	*rhlE*	STM0820	ATP-dependent RNA helicase RhlE	Ribosome	CecR activated
-1.37	-0.89	-0.24	-1.49	1.12	-0.60	RS20200	*wecF*	*rfe-wzzE-wecBC-rffGHC-wecE-wzxE-wecF-wzyE-rffM-yifK*	STM3927	TDP-N-acetylfucosamine:lipid II N-acetylfucosaminyltransferase	Envelope - ECA synthesis	NsrR repressed
-2.31	-2.46	-1.19	-2.34	1.11	0.12	RS14845	*sirC*	SPI-1	STM2867	Transcriptional regulator	SPI-1	
-1.61	-1.00	-0.50	-1.80	1.11	-0.80	RS22535	RS22535	RS22535	STM4390	Hypothetical protein	Unknown	
-3.60	-0.95	-2.49	-1.70	1.11	-0.75	RS09310	RS09310	RS09310-5	STM1863	Putative DUF5993 family protein	Unknown	

-1.58	0.40 †	-0.47	-0.85	1.11	-1.25	RS21040	*priA*	*priA*	STM4095	primosomal protein N	DNA replication	
-1.61	-2.50	-0.50	-3.14	1.10	-0.64	RS17760	*rplC*	*rpsJ-rplCDWB-rpsS-rplV-rpsC-rplP-rpmC-rpsQ*	STM3440	50S ribosomal protein L3	Ribosome	ppGpp / DksA repressed
-1.30	0.16 †	-0.21	-1.00	1.08	-1.15	RS13220	*era*	*rnc-era-recO-pdxJ-acpS*	STM2580	GTPase Era	Ribosome	
-1.47	0.03 †	-0.39	-1.00	1.08	-1.03	RS11145	*pbpG*	*pbpG*	STM2168	D-alanyl-D-alanine endopeptidase (PBP7)	Envelope - Peptidoglycan synthesis / modification	
-1.52	-2.38	-0.44	-3.26	1.08	-0.87	RS17645	*rpsK*	*rpsMKD-rpoA*	STM3417	30S ribosomal protein S11	Ribosome	ppGpp / DksA repressed in coli
-1.34	-0.44 †	-0.26	-1.38	1.08	-0.94	RS18025	*mrcA*	*mrcA*	STM3493	Peptidoglycan glycosyltransferase/peptidoglycan DD-transpeptidase (PBP1a)	Envelope - Peptidoglycan synthesis / modification	
-1.73	1.21	-0.65	0.45	1.08	-0.76	RS04350	RS04350	RS04350	STM0859	Putative LysR family transcriptional regulator	Regulation	
-1.39	0.82	-0.31	-0.72	1.08	-1.53	RS13620	*pssA*	*pssA*	STM2652	CDP-diacylglycerol--serine O-phosphatidyltransferase	Envelope - Phospholipid synthesis	
-1.41	-1.00	-0.34	-1.87	1.07	-0.86	RS16980	*rimP*	*rimP-nusA-infB*	STM3288	Ribosome maturation factor	Ribosome	Fis activated, Nac, ArgR, CRP repressed
-1.52	-0.08 †	-0.46	-1.23	1.07	-1.15	RS08255	*ttcA*	RS08250-*ttcA*	STM1654	tRNA 2-thiocytidine(32) synthetase	tRNA & related	
-1.66	-0.11 †	-0.60	-1.51	1.06	-1.40	RS15635	RS15635	RS15635	STM3022	Putative amino acid permease	AA metabolism - Amino acid transport	
-1.73	-1.96	-0.68	-2.11	1.05	-0.15	RS22150	RS22150	RS22150-22145-26870-22135	STM4315	AraC family transcriptional regulator	Unknown - Operon is regulatory target of HilD (1)	
-1.68	0.35	-0.64	-0.77	1.04	-1.12	RS00445	*folA*	*folA*	STM0087	Type 3 dihydrofolate reductase	Tetrahydrofolate biosynthesis	IHF, TyrR activated
-3.22	1.02	-2.18	-0.20 †	1.04	-1.22	RS03005	*entC*	*entCEBAH*	STM0595	Isochorismate synthase	Iron acquisition	CRP activated, Fur repressed
-2.22	-1.72	-1.18	-1.91	1.04	-0.20	RS14850	*orgC*	SPI-1	STM2868	Type III secretion system effector protein	SPI-1	
-2.48	-1.48	-1.45	-1.64	1.03	-0.16	RS14985	*spaK*	SPI-1	STM2895	SPI-1 type III secretion system chaperone	SPI-1	
-1.57	0.30 †	-0.54	-0.90	1.03	-1.20	RS06175	tRNA0022	tRNA0022	0	tRNA-Arg	tRNA & related	
-1.32	0.08 †	-0.29	-0.97	1.03	-1.05	RS20445	*rfaH*	*rfaH*	STM3977	Transcription/translation regulatory transformer protein	Envelope – LPS synthesis	Nac activated
-1.53	-2.01	-0.50	-2.73	1.03	-0.72	RS17710	*rplN*	*rplNXE-rpsNH-rplFR-rpsE-rpmD-rp10-secY-rpmJ*	STM3430	50S ribosomal protein L14	Ribosome	ppGpp / DksA repressed in coli
-2.42	2.23	-1.39	1.46	1.03	-0.77	RS23105	RS23105	RS23105	STM4504	Hypothetical protein	Unknown	
-1.75	4.70	-0.73	3.20	1.02	-1.51	RS21000	*glpF*	*glpFKX*	STM4087	Glycerol facilitator	Glycerol uptake	GRP activated, GlpR repressed
-1.77	-0.18 †	-0.76	-1.25	1.02	-1.06	RS17035	*yhbY*	*yhbY*	STM3298	Ribosome assembly factor	Ribosome	Lrp repressed
-1.30	-0.94	-0.28	-1.57	1.02	-0.63	RS01160	*lpxB*	*bamA-skp-lpxD-fabZ-lpxAB-rnhA-dnaE-accA*	STM0229	Lipid A disaccharide synthase	Envelope – LPS synthesis	σ^{24} activated
-1.24	0.20 †	-0.23	-1.05	1.02	-1.25	RS13575	*srmB*	*srmB*	STM2643	ATP-dependent RNA helicase	Ribosome	
-1.58	0.94	-0.57	-0.03 †	1.01	-0.97	RS05475	*scsB*	RS05470-80-*agp*	STM1114	Protein-disulphide reductase	Disulphide bond formation	
-1.30	-1.47	-0.30	-2.41	1.00	-0.94	RS05245	*lonH*	*lonH*	STM1068	Lon protease family protein		

Key to columns (left to right)

Log_2 fold change values for each gene: 1 h vs 3 h in the wild type; 3 h vs 5 h in the wild type; 1 h vs 3 h in Δ*acrB*; 3 h vs 5 h in Δ*acrB*.

ΔLog$_2$ values for the 1 h vs 3 h and 3 h vs 5 h comparisons. Δlog$_2$ values are calculated by Log$_2$ fold change for Δ*acrB* minus Log$_2$ fold change for the wild type. Positive values indicate more upregulation or less downregulation in wt, negative values more upregulation or less downregulation in *acrB*. Genes are sorted in decreasing Δlog$_2$ 1vs3 h order.

ID: locus name.

Gene name

Operon structure: if unknown are inferred from genomic sequence and comparison with ecocyc.com.

STM: STM gene number.

Product: Function of gene product, if known.

Comments: General classification of function of gene product.

Regulation in *E. coli*: sourced from Ecocyc.com unless otherwise referenced.

Colour mapping:

-4	-3	-2	-1	0	1	2	3	4

Log$_2$ fold change values that are non-significant (p_{adj} > 0.05) are in white and marked with a dagger †. ΔLog$_2$ fold change values derived from one or more non-significant Log$_2$ fold change values are also in white.

Supplemental Table S2. Genes in group two - Genes that are more upregulated at 3hr in Δ*acrB* than wt – "early activation" pattern

Log$_2$ fold change				Δ*acrB* - wt Δlog$_2$										
**wt 1vs3**	**wt 3vs5**	**Δ*acrB* 1vs3**	**Δ*acrB* 3vs5**	**1vs3**	**3vs5**	**ID**	**Gene name**	**Operon**	**STM**	**Product**	**Comments**	**Regulation in *Salmonella***	**Regulation in *E. coli***	**SalComMac log$_2$ fold change Anaerobic shock**
0.84	3.50	3.85	0.08 †	3.01	-3.41	SL1344_RS05365	*orfX*	*orfX*	STM1092	Hypothetical YlcI/YnfO family protein	Part of SPI-5	DksA repressed (3)		5.23
2.12	-1.18	4.32	-2.21	2.20	-1.03	SL1344_RS06235	*yntB / nikB*	*yntABCDE / nikABCDE*	STM1256	ABC transporter permease	High-affinity nickel transporter		FNR activated, NikR repressed	2.25
1.02	-0.79 †	3.18	-2.17	2.15	-1.38	SL1344_RS06240	*yntC / nikC*	*yntABCDE / nikABCDE*	STM1257	ABC transporter permease	High-affinity nickel transporter		FNR activated, NikR repressed	1.93
0.82	-0.75	2.93	-1.81	2.11	-1.05	SL1344_RS06250	*yntE / nikE*	*yntABCDE / nikABCDE*	STM1259	Peptide ABC transporter ATP-binding protein	High-affinity nickel transporter		FNR activated, NikR repressed	1.68
3.40	-2.06	5.40	-3.19	2.00	-1.14	SL1344_RS06230	*yntA / nikA*	*yntABCDE / nikABCDE*	STM1255	Nickel ABC transporter substrate-binding protein	High-affinity nickel transporter		FNR activated, NikR repressed	4.09
-0.62	-1.78	1.29	-3.26	1.91	-1.47	SL1344_RS19800		19800- 19805	STM3845	Hypothetical protein (retron St85 family effector protein)	Anti-phage defence			-1.25
-0.66	1.24	1.03	-0.31 †	1.69	-1.56	SL1344_RS16765	*tdcB*	*tdcABCD-pflB-tdcG*	STM3244	Bifunctional threonine ammonia-lyase/L-serine ammonia-lyase	Anaerobic serine / threonine degradation	H-NS repressed, TdcA activated (4)	FNR, CRP, IHF, TdcA, TdcR activated	7.45
-0.89	0.64 †	0.79	-0.72	1.68	-1.35	SL1344_RS09910	*fliR*	*fliLMNOPQR*	STM1981	Flagellar type III secretion system protein	Flagella - Class 2 flagellar gene		σ^{70}/ σ^{28}, FlhDC activated	-2.12
1.79	0.27 †	3.46	-1.91	1.67	-2.18	SL1344_RS15890		15890-15910	STM3071	Putative DNA-binding protein	Putative Co^{2+} / Ni^{2+} transporter			2.14
-0.49	0.23 †	1.14	-2.11	1.62	-2.34	SL1344_RS09690	*tyrP*	*tyrP*	STM1937	Tyrosine transporter TyrP	Tyrosine import		TyrR, Lrp regulated, IHF repressed	-1.51
1.49	-0.53 †	3.04	-2.28	1.55	-1.75	SL1344_RS11590	*napB*	*napFDAGHBC-ccmABCDEFG2*	STM2256	nitrate reductase cytochrome c-type subunit	Anaerobic respiration – Periplasmic nitrate reductase		FNR, ModE,FlhCD activated, MArP regulated, NarL, IscR repressed	4.69
-0.77	-0.19 †	0.77	-1.41	1.53	-1.22	SL1344_RS14220		14220	STM2747	DUF4435 domain-containing protein	Unknown function			-0.18
2.45	-1.51	3.95	-2.30	1.50	-0.79	SL1344_RS06245	*yntD / nikD*	*yntABCDE / nikABCDE*	STM1258	ATP-binding cassette domain-containing protein	High-affinity nickel transporter		FNR activated, NikR repressed	1.74
2.11	-0.63 †	3.59	-2.68	1.48	-2.05	SL1344_RS11600	*napG*	*napFDAGHBC-ccmABCDEFG2*	STM2258	ferredoxin-type protein NapG	Anaerobic respiration – Periplasmic nitrate reductase		FNR, ModE,FlhCD activated, NarP regulated, NarL, IscR repressed	4.78
-0.66	3.84	0.81	2.43	1.46	-1.41	SL1344_RS11710		11710	STM2281	LysR family transcriptional regulator	Unknown function			1.92
1.81	-1.12	3.26	-2.37	1.45	-1.25	SL1344_RS11560	*ccmE2*	*napFDAGHBC-ccmABCDEFG2*	STM2250	cytochrome c maturation protein CcmE	Cytochrome *c* maturation – cluster 2		FNR, ModE, FlhCD activated, NarP regulated, NarL, IscR repressed	0.32
2.60	0.96	4.05	-0.25 †	1.45	-1.21	SL1344_RS19665	*yhjA / ccp*	*ccp-?-ccmABSCDEFG1*	STM3820	c-type cytochrome	Cytochrome *c* peroxidase	FNR activated(5)	FNR, OxyR activated	0.03
-0.93	3.58	0.41	2.21	1.34	-1.38	SL1344_RS19740		19740	STM3834	LysR family transcriptional regulator	Unknown function			3.13
-0.85	-0.53 †	0.48	-2.18	1.33	-1.64	SL1344_RS15045	*pphB*	*pphB*	STM2907	Serine/threonine protein phosphatase	SPI-1		Nac repressed	-0.79
1.89	-0.97 †	3.21	-2.68	1.32	-1.71	SL1344_RS11605	*napA*	*napFDAGHBC-ccmABCDEFG2*	STM2259	nitrate reductase catalytic subunit NapA	Anaerobic respiration – Periplasmic nitrate reductase		FNR, ModE,FlhCD activated, NarP regulated, NarL, IscR repressed	5.38
2.13	-1.39	3.45	-2.46	1.32	-1.07	SL1344_RS11555	*ccmF2*	*napFDAGHBC-ccmABCDEFG2*	STM2249	c-type cytochrome biogenesis protein CcmF	Cytochrome *c* maturation – cluster 2		FNR, ModE,FlhCD activated, NarP regulated, NarL, IscR repressed	0.00
0.73	-3.01	2.04	-3.07	1.32	-0.06	SL1344_RS09850	*fliF*	*fliFGHIJKL*	STM1969	flagellar M-ring protein FliF	Flagella - Class 2 flagellar gene	FNR, FlhDC activated (5, 6)	σ^{70}/ σ^{28}, FlhDC activated, CsgD repressed	-2.64
2.00	0.50 †	3.31	-1.31	1.32	-1.81	SL1344_RS21950	*nrfA*	*nrfABCDEFG*	STM4277	ammonia-forming nitrite reductase cytochrome c552 subunit	Anaerobic respiration – Periplasmic nitrite reductase	FNR activated (5)	FNR,FlhDC, NarP activated, IHF, NarL regulated, Fis, NsrR repressed	5.42
-0.58	0.18 †	0.69	-0.88	1.27	-1.06	SL1344_RS03870		03870	STM0764	LysR family transcriptional regulator	Unknown function			0.00

0.70	-0.73 †	1.95	-1.83	1.25	-1.10	SL1344_RS09685	*yecH*	*yecH*	STM1936	Hypothetical protein	Unknown function – divergent from *tyrP*	Downregulated by adrenaline (7)		2.74
-0.69	-0.47	0.55	-1.48	1.24	-1.01	SL1344_RS11335	*setB*	*setB*	STM2207	Sugar efflux transporter SetB	Sugar efflux			0.00
-0.91	0.37 †	0.33	-0.58	1.24	-0.95	SL1344_RS13240	*gogB*	*gogB*	STM2584	Phage-encoded type III secretion effector GogB	Phage			-0.29
0.80	-1.19	2.03	-1.89	1.23	-0.70	SL1344_RS07940	*srfA*	*srfABC*	STM1593	SsrAB-activated protein	Proposed virulence effector; class 2 flagellar gene	FNR, FlhDC activated (5, 6)		-2.47
1.34	-1.64	2.56	-2.57	1.22	-0.93	SL1344_RS11545	*ccmH2*	*napFDAGHBC-ccmABCDEFG2*	STM2247	cytochrome c-type biogenesis protein CcmH	Cytochrome *c* maturation – cluster 2		FNR, ModE,FlhCD activated, NarP regulated, NarL, IscR repressed	2.41
2.05	-1.55	3.26	-2.08	1.22	-0.52	SL1344_RS11565	*ccmD2*	*napFDAGHBC-ccmABCDEFG2*	STM2251	heme exporter protein CcmD	Cytochrome *c* maturation – cluster 2		FNR, ModE,FlhCD activated, NarP regulated, NarL, IscR repressed	0.00
1.86	-0.72 †	3.06	-1.93	1.20	-1.20	SL1344_RS19660	*ccmA1*	*ccp-?-ccmABSCDEFGH1*	STM3819	cytochrome c biogenesis heme-transporting ATPase CcmA	Cytochrome *c* maturation – cluster 1			
1.56	-0.63 †	2.76	-2.02	1.20	-1.39	SL1344_RS19655	*ccmB1*	*ccp-?-ccmABSCDEFGH1*	STM3818	heme exporter protein CcmB	Cytochrome *c* maturation – cluster 1			0.00
1.65	-0.58 †	2.85	-2.28	1.20	-1.70	SL1344_RS11575	*ccmB2*	*napFDAGHBC-ccmABCDEFG2*	STM2253	heme exporter protein CcmB	Cytochrome *c* maturation – cluster 2		FNR, ModE,FlhCD activated, NarP regulated, NarL, IscR repressed	0.00
-0.47	-0.41 †	0.72	-1.18	1.19	-0.77	SL1344_RS23215	*hsdS*	*hsdMS*	STM4524	restriction endonuclease subunit S	Restriction endonuclease			0.26
2.26	2.32	3.43	0.80 †	1.17	-1.52	SL1344_RS22105		22105-22120	STM4305	molybdopterin-dependent oxidoreductase	Putative DMSO reductase	FNR activated (5)		4.01
-0.74	5.35	0.43	3.70	1.17	-1.65	SL1344_RS20930	*lsrR*	*lsrRK*	STM4073	transcriptional regulator	Quorum sensing		CRP activated, LsrR repressed	-0.36
2.03	-0.72 †	3.19	-2.03	1.16	-1.31	SL1344_RS11580	*ccmA2*	*napFDAGHBC-ccmABCDEFG2*	STM2254	cytochrome c biogenesis heme-transporting ATPase CcmA	Cytochrome *c* maturation – cluster 2		FNR, ModE,FlhCD activated, NarP regulated, NarL, IscR repressed	0.00
3.01	-0.53 †	4.15	-1.78	1.14	-1.24	SL1344_RS16910	*yhbU / ubiU*	*ubiUV*	STM3274	O_2-independent ubiquinone biosynthesis protein	Ubiquinone biosynthesis	FNR activated (5)	Nac repressed	3.57
1.86	-0.23 †	2.99	-1.50	1.13	-1.27	SL1344_RS15900	*ecfT*	15890-15910	STM3073	Energy-coupling factor transporter transmembrane protein EcfT - cobalt transporter?	Putative Co^{2+} / Ni^{2+} transporter			1.29
-0.86	-0.35 †	0.26	-1.40	1.12	-1.05	SL1344_RS08880	*ispE / ipk*	*lolB-ispE*	STM1779	4-(cytidine 5'-diphospho)-2-C-methyl-D-erythritol kinase	Isoprenoid synthesis pathway			-1.64
0.74	4.78	1.85	3.40 †	1.10	-1.38	SL1344_RS12010		12020-12005	STM2341	Putative transketolase	Unknown function	FNR repressed (5)		4.45
-0.48	3.56	0.61	2.48	1.09	-1.08	SL1344_RS21085		21085	STM4103	Hypothetical protein	Unknown function			1.63
2.14	-1.46	3.21	-2.52	1.07	-1.06	SL1344_RS19635	*ccmF1*	*ccp-?-ccmABSCDEFGH1*	STM3814	*c*-type cytochrome biogenesis protein CcmF	Cytochrome *c* maturation – cluster 1			0.00
1.99	-0.82	3.04	-1.90	1.05	-1.08	SL1344_RS19640	*ccmE1*	*ccp-?-ccmABSCDEFGH1*	STM3815	Cytochrome c maturation protein CcmE	Cytochrome *c* maturation – cluster 1			0.32
1.01	-3.72	2.03	-2.85	1.02	0.86	SL1344_RS09860	*fliH*	*fliFGHIJKL*	STM1971	Flagellar assembly protein FliH	Flagella - Class 2 flagellar gene	FNR, FlhDC activated (5, 6)	σ^{70}/ σ^{28}, FlhDC activated, CsgD repressed	-2.56
1.79	-1.27	2.79	-2.88	1.00	-1.61	SL1344_RS11550	*ccmG2*	*napFDAGHBC-ccmABCDEFG2*	STM2248	Thiol:disulfide interchange protein	Cytochrome *c* maturation – cluster 2		FNR, ModE,FlhCD activated, NarP regulated, NarL, IscR repressed	0.00

Key to columns (left to right)

Log_2 fold change values for each gene: 1 h vs 3 h in the wild type; 3 h vs 5 h in the wild type; 1 h vs 3 h in Δ*acrB*; 3 h vs 5 h in Δ*acrB*.

ΔLog_2 values for the 1 h vs 3 h and 3 h vs 5 h comparisons. Δlog_2 values are calculated by Log_2 fold change for Δ*acrB* minus Log_2 fold change for the wild type. Positive values indicate more upregulation or less downregulation in wt, negative values more upregulation or less downregulation in *acrB*. Genes are sorted in decreasing Δlog_2 1vs3 h order.

ID: locus name.
Gene name
Operon structure: if unknown are inferred from genomic sequence and comparison with ecocyc.com.
STM: STM gene number.
Product: Function of gene product, if known.
Comments: General classification of function of gene product.
Regulation in *Salmonella*: known regulation, as referenced.
Regulation in *E. coli*: sourced from Ecocyc.com unless otherwise referenced.
SalComMac log_2 Anaerobic shock: Gene expression data from SalComMac (http://bioinf.gen.tcd.ie/cgi-bin/salcom.pl?db=salcom_mac_HL) for the anaerobic shock condition; "Growth in Lennox broth to OD_{600} 0.3 (50 ml), then filled into 50 ml closed Falcon tube and incubated without agitation at 37°C for 30 min." (8).

Colour mapping:

-4	-3	-2	-1	0	1	2	3	4

Log_2 fold change values that are non-significant (p_{adj} > 0.05) are in white and marked with a dagger †. ΔLog_2 fold change values derived from one or more non-significant Log_2 fold change values are also in white.

Supplemental Table S3. Genes in group three - Genes that are more upregulated at 3hr in *wt* than *acrB*

Log_2 fold change				Δ*acrB* – wt Δlog_2								
**wt 1vs3**	**wt 3vs5**	**Δ*acrB* 1vs3**	**Δ*acrB* 3vs5**	**1vs3**	**3vs5**	**ID**	**Gene**	**Operon**	**STM**	**Product**	**Comments**	**Regulation in *E. coli***
7.28	-2.01	2.33	2.80	-4.95	4.81	SL1344_RS21095	*metF*	*metF*	STM4105	Methylenetetrahydrofolate reductase	Methionine / SAM synthesis	MetJ repressed
5.70	-3.19	0.77	1.46	-4.93	4.64	SL1344_RS20380	*metR*	*metR*	STM3964	HTH-type transcriptional regulator MetR	Methionine / SAM synthesis	MetJ, MetR repressed
7.88	-1.84	3.10	3.65	-4.78	5.49	SL1344_RS20385	*metE*	*metE*	STM3965	5 - methyltetrahydropteroyltriglutamate homocysteine S-methyltransferase	Methionine / SAM synthesis	MetJ repressed, MetR, OxyR activated
5.42	-1.62	1.00	2.79	-4.42	4.40	SL1344_RS21470	*metA*	*metA*	STM4182	Homoserine O-succinyltransferase	Methionine / SAM synthesis	MetJ repressed
4.79	-3.08	0.44	1.50	-4.35	4.58	SL1344_RS21070	*metL*	*metBL*	STM4101	Bifunctional aspartate kinase/homoserine dehydrogenase II	Methionine / SAM synthesis	MetJ repressed, PhoP activated
6.20	-3.28	1.87	1.61	-4.33	4.88	SL1344_RS03045	*ybdL/ mtnE*	*ybdL/mtnE*	STM0603	Methionine aminotransferase	Methionine salvage pathway from polyamine biosynthesis pathway.	Lrp repressed<br>MetJ regulated? (9)
4.74	-3.74	0.44	0.96	-4.30	4.70	SL1344_RS21075	*(mscS)*		STM4102	Pseudogene - mechanosensitive ion channel	Pseudogene, contains a stop codon	
5.47	-5.17	1.46	-0.62	-4.01	4.55	SL1344_RS19860	*pstS*	*pstSCAB-phoU*	STM3857	Phosphate ABC transporter substrate-binding protein PstS	High affinity phosphate uptake	RpoS,FNR,IHF,PhoB activated, Nac repressed
5.91	-2.37	1.91	2.02	-4.00	4.39	SL1344_RS03040	*ybdH*	*ybdH*	STM0602	Putative glycerol dehydrogenase	Glycerol utilisation?	MetJ regulated? (9)
4.74	-3.44	1.23	-0.26 †	-3.51	3.18	SL1344_RS19850	*pstA*	*pstSCAB-phoU*	STM3855	Phosphate ABC transporter permease PstA	High affinity phosphate uptake	RpoS,FNR,IHF,PhoB activated, Nac repressed
4.50	-3.37	1.17	-0.16 †	-3.32	3.21	SL1344_RS19855	*pstC*	*pstSCAB-phoU*	STM3856	Phosphate ABC transporter permease PstC	High affinity phosphate uptake	RpoS,FNR,IHF,PhoB activated, Nac repressed
3.65	-0.12 †	0.51	2.29	-3.14	2.41	SL1344_RS03035	*ybdD*	*ybdD*	STM0601	YbdD/YjiX family protein	Pyruvate import in *E. coli*	
4.12	-2.17	1.02	0.76	-3.10	2.92	SL1344_RS01250	*metN*	*metNIQ*	STM0247	Methionine ABC transporter ATP-binding protein MetN	Methionine uptake / SAM synthesis	MetJ repressed, HypT activated
4.00	-3.50	1.16	-0.52	-2.85	2.98	SL1344_RS19845	*pstB*	*pstSCAB-phoU*	STM3854	Phosphate ABC transporter ATP-binding protein PstB	High affinity phosphate uptake	RpoS,FNR,IHF,PhoB activated, Nac repressed
3.83	-1.88	1.07	1.14	-2.76	3.02	SL1344_RS03050	*ybdM*	*ybdNM*	STM0604	Transcriptional regulator related to SpoOJ (10)	Function unknown	
1.62	-0.95	-1.01	1.20	-2.63	2.15	SL1344_RS22020	*proP*	*proP*	STM4290	Proline / glycine betaine transporter	Osmotic stress response	RpoS, Lrp, Fis activated
3.42	-1.33	0.90	0.85	-2.52	2.18	SL1344_RS16345	*metC*	*metC*	STM3161	Cystathionine beta-lyase	Methionine / SAM synthesis	MetJ repressed
3.59	-1.43	1.26	1.02	-2.32	2.45	SL1344_RS01245	*metI*	*metNIQ*	STM0246	Methionine ABC transporter permease MetI	Methionine uptake / SAM synthesis	MetJ repressed, HypT activated
3.47	-0.74 †	1.26	1.08	-2.21	1.83	SL1344_RS21100	*katG*	*katG*	STM4106	Catalase/peroxidase HPI	ROS defence - catalase	Induced on entry into stationary phase (11)
2.49	-0.26 †	0.48	1.83	-2.02	2.10	SL1344_RS21475	*aceB*	*aceBAK*	STM4183	Malate synthase A	Glyoxylate shunt	Lrp, Cra, IHF activated, ArcA, Nac, CRP, IclR repressed
1.11	-0.92	-0.73	1.08	-1.84	2.00	SL1344_RS04365	*yliJ / gstB*	*yliJ*	STM0862	Putative glutathione S-transferase	Dehalogenates Br-acetate and I-acetate in *E. coli*	
3.27	-0.87 †	1.45	0.57 †	-1.82	1.44	SL1344_RS25905	*yqgD*	*yqgD*	STM3089	Hypothetical protein	Function unknown	

1.24	0.92 †	-0.50	2.05	-1.74	1.13	SL1344_RS05680	*msyB*	*msyB*	STM1153	SecY/SecA suppressor protein	Periplasmic protein export	RpoS activated (12)
0.98	0.35 †	-0.69	1.59	-1.67	1.24	SL1344_RS21380	*rsd*	*rsd*	STM4165	Anti-RNA polymerase sigma 70 factor	Coordinates entry to stationary phase.	Lrp,McbR,RcdA,SdiA,SlyA activated, ArcA Nac repressed
-0.83	0.00 †	-2.45	0.00 †	-1.62	0.00	SL1344_RS14535	*nrdI*	*nrdHI*	STM2806	Ribonucleotide reductase assembly protein NrdI	Nucelotide metabolism	IscR activated, Fur and NrdR repressed
1.10	2.36	-0.47	2.99	-1.58	0.63	SL1344_RS01875	*yahO*	*yahO*	STM0366	Hypothetical protein	Function unknown	RpoS, ppGpp activated
3.46	-0.81 †	1.89	0.99	-1.57	1.80	SL1344_RS15985	*metK*	*metK*	STM3090	Methionine adenosyltransferase	SAM synthesis	MetJ repressed, CRP repressed
1.03	0.64 †	-0.53	1.46	-1.56	0.82	SL1344_RS05675	*yceK*	*yceK*	STM1152	Hypothetical OM lipoprotein	LPS assembly in *E. coli* (13)	RpoS activated (12)
1.89	-0.93 †	0.43	1.13	-1.46	2.06	SL1344_RS02595	*sfbA*	*sfbABC*	STM0510	Metal ABC transporter substrate-binding protein	Iron uptake?	
1.80	0.13 †	0.35	2.03	-1.45	1.90	SL1344_RS11210	*yohK*	*yohJK*	STM2182	Putative 3-hydroxypropanoate export protein	Hydroxypropanoate export?	
0.93	0.62 †	-0.45	1.55	-1.38	0.93	SL1344_RS10650	*yeeZ*	*yeeZ*	STM2070	NAD(P)-dependent oxidoreductase	Unknown function	
0.95	-0.12 †	-0.42	0.98	-1.37	1.09	SL1344_RS00815	*yacL*	*yacL*	STM0160	Protein YacL	Unknown function	Lrp activated
0.91	0.01 †	-0.40	1.41	-1.31	1.41	SL1344_RS04590	*clpA*	*clpSA*	STM0945	ATP-dependent Clp protease ATP-binding subunit	Proteolysis	
1.68	-0.62 †	0.39	0.47 †	-1.29	1.10	SL1344_RS11205	*yohJ*	*yohJK*	STM2181	Putative 3-hydroxypropanoate export protein	Hydroxypropanoate export?	
1.60	0.18 †	0.34	1.34	-1.26	1.16	SL1344_RS06585	*ghoS*	*ghoS*	STM1326	Type V toxin-antitoxin system endoribonuclease antitoxin GhoS	TA system	
2.30	3.74	1.05	4.61	-1.25	0.87	SL1344_RS05235	*rmf*	*rmf*	STM1066	Ribosome modulation factor	Converts ribosomes to dimeric form in stationary phase	ppGpp activated, ArcA repressed
1.70	1.86	0.47	2.41	-1.23	0.55	SL1344_RS06940	*sseA*	SPI-2	STM1397	SPI-2 type III secretion system chaperone SseA	SPI-2	
0.77	-1.13	-0.46	-0.02 †	-1.23	1.11	SL1344_RS15870	*yggB / mscS*	*yggB*	STM3067	small-conductance mechanosensitive channel MscS	Mechanosensing	Lrp repressed
1.61	1.69	0.40	2.19	-1.21	0.50	SL1344_RS17230	*yhcH / nanQ*	*nanTEKQ*	STM3335	N-acetylneuraminate anomerase	Sialic acid metabolism	CRP activated, Fis, NanR repressed
2.30	0.69	1.09	2.01	-1.20	1.32	SL1344_RS18435	*tcp*	*tcp*	STM3577	Methyl-accepting chemotaxis protein II	Chemotaxis	
2.89	-1.36	1.69	-0.52	-1.20	0.84	SL1344_RS16195	*hcp*	*hcp-hcr*	STM3131	Type VI secretion system tube protein Hcp	Protein secretion	
0.81	0.58 †	-0.36	1.17	-1.17	0.59	SL1344_RS06900	*orf319*	SPI-2	STM1389	hypothetical protein	SPI-2	
1.51	-1.05	0.34	0.49	-1.17	1.54	SL1344_RS18795	*ghrB*	*ghrB*	STM3646	Glyoxylate / hydroxypyruvate reductase	Gluconate metabolism	
1.65	-0.31 †	0.51	0.91	-1.14	1.21	SL1344_RS11875	*cheV*	*cheV*	STM2314	Chemotaxis protein CheV	Chemotaxis	
1.58	1.28	0.45	1.70	-1.13	0.43	SL1344_RS06995	*ssaI*	SPI-2	STM1408	EscI/YscI/HrpB family type III secretion system inner rod protein	SPI-2	
0.85	-0.33 †	-0.27	1.16	-1.12	1.48	SL1344_RS04585	*clpS*	*clpSA*	STM0944	ATP-dependent Clp protease adapter ClpS	Proteolysis	Lrp activated, PhoP repressed
0.69	0.19 †	-0.41	1.17	-1.09	0.98	SL1344_RS19370	*cigR*	*cigR*	STM3762	Anti-virulence regulatory inner membrane protein	Regulator of virulence	
0.73	0.72 †	-0.35	0.95	-1.07	0.23	SL1344_RS06575	*yniB*	*yniB*	STM1323	Hypothetical protein	Unknown function	
1.59	0.29 †	0.52	1.18	-1.07	0.88	SL1344_RS02190	*phnT*	*phnSTUV*	STM0428	2-aminoethylphosphonate ABC transport system ATP-binding subunit PhnT	Aminoethylphosphonate import	
2.24	-0.31 †	1.18	0.63	-1.06	0.93	SL1344_RS18505	*uspA*	*uspA*	STM3591	Universal stress protein UspA	Stress response	IHF, ppGpp activated, Nac, FadR repressed

1.60	1.25	0.54	1.55	-1.06	0.30	SL1344_RS06990	*ssaH*	SPI-2		EscG/YscG/SsaH family type III secretion system needle protein co-chaperone	SPI-2, HilA repressed (14)	
0.83	-0.04 †	-0.23	0.58	-1.06	0.62	SL1344_RS12340		RS12340	STM2404	Ion channel protein		
0.57	0.56	-0.43	0.89	-1.00	0.33	SL1344_RS18425	*yhhN*	*yhhN*	STM3575	Hypothetical protein	Unknown function	

Key to columns (left to right)

Log_2 fold change values for each gene: 1 h vs 3 h in the wild type; 3 h vs 5 h in the wild type; 1 h vs 3 h in Δ*acrB*; 3 h vs 5 h in Δ*acrB*.

ΔLog_2 values for the 1 h vs 3 h and 3 h vs 5 h comparisons. Δlog_2 values are calculated by Log_2 fold change for Δ*acrB* minus Log_2 fold change for the wild type. Positive values indicate more upregulation or less downregulation in wt, negative values more upregulation or less downregulation in *acrB*. Genes are sorted in decreasing Δlog_2 1vs3 h order.

ID: locus name.

Gene name

Operon structure: if unknown are inferred from genomic sequence and comparison with ecocyc.com.

STM: STM gene number.

Product: Function of gene product, if known.

Comments: General classification of function of gene product.

Regulation in *E. coli*: sourced from Ecocyc.com unless otherwise referenced.

Colour mapping:

-4	-3	-2	-1	0	1	2	3	4

Log_2 fold change values that are non-significant ($p_{adj} > 0.05$) are in white and marked with a dagger †. ΔLog_2 fold change values derived from one or more non-significant Log_2 fold change values are also in white.

Supplementary Table S4. Antimicrobial susceptibility of wild-type, AcrB-inactivated, and AcrAB-overexpressing strains.

	MIC (µg·mL^{-1})																				
**Strain**	**NAF**	**OXA**	**CEF**	**NAL**	**CIP**	**ERY**	**AZT**	**EtBr**	**FA**	**NOV**	**APR**	**GEN**	**TOB**	**SPC**	**HYG**	**STR**	**TET**	**CHL**	**RIF**	**AZM**	**PB**
***S.* Typhimurium**																					
SL1344 (WT)	>1024	512	0.125	4	0.016	128	4	>1024	1024	128	2	0.5	2	32	32	8	1	2	8	0.06	2
SL1344 Δ*acrB*	16	<1	0.03	1	<0.008	2	0.5	64	8	4	2	0.5	2	32	16	8	0.5	0.5	4	0.06	2
SL1344 *acrB* D408A	8	<1	0.03	1	<0.008	2	0.5	32	4	2	2	0.5	1	32	16	8	0.5	0.5	4	0.06	4
SL1344 + pPAR1-S*acrAB*	>1024	512	0.125	4	0.016	128	4	>1024	1024	256	2	0.5	4	32	32	8	1	4	8	0.06	2
SL1344 Δ*acrB* + pPAR1-S*acrAB*	1024	256	0.06	4	0.016	64	2	1024	256	128	2	0.5	4	32	32	8	0.5	2	4	0.06	2
E. coli																					
MG1655 (WT)	1024	256	0.06	8	0.008	64	2	1024	512	128	<1	0.25	0.5	8	8	2	1	4	8	0.06	0.5
MG1655 Δ*acrB*	8	2	0.016	2	<0.008	4	0.5	16	8	4	<1	0.125	0.5	4	8	2	0.25	0.5	4	0.06	0.5
MG1655 *acrB* D408A	8	2	0.016	2	<0.008	4	0.5	16	8	4	<1	0.125	0.25	4	8	2	0.25	0.5	4	0.06	1
MG1655 + pPAR7-E*acrAB*	Amp^{R}	Amp^{R}	0.06	8	0.008	32	2	1024	512	128	<1	0.125	0.25	4	8	2	1	4	4	0.06	0.5
MG1655 Δ*acrB* + pPAR7-E*acrAB*	Amp^{R}	Amp^{R}	0.03	4	<0.008	32	2	256	256	32	<1	0.125	0.25	4	8	2	1	4	4	0.06	0.5

Minimum inhibitory concentrations (MIC) for a range of antimicrobials was determined using an agar dilution MIC assay.

NAF – nafcillin; OXA – oxacillin; CEF – cefotaxime; NAL – nalidixic acid; CIP – ciprofloxacin; ERY – erythromycin; AZT – azithromycin; EtBr – ethidium bromide; FA – fusidic acid; NOV – novobiocin; APR – apramycin; GEN – gentamicin; TOB – tobramycin; SPC – spectinomycin; HYG – hygromycin; STR – streptomycin; TET – tetracycline; CHL – chloramphenicol; RIF – rifampicin; AZM – aztreonam; PB – polymyxin B.

Amp^{R} – pPAR7-E*acrAB* contains an ampicillin resistance cassette and therefore transformants are ampicillin resistant.

Supplementary Table S5. Plasmids and oligonucleotide primers used in this study.

**Plasmids**		
**Name**	**Description**	**Source**
pBAD30	p15A origin, Amp^R, arabinose-inducible expression plasmid.	(15)
pET26b	pBR322 origin, Kan^R, T7 expression plasmid. Source of Kan^R.	Novagen
pBAD30-*kanR*	p15A origin, Kan^R, arabinose-inducible expression plasmid.	This study
p15A-lacI	p15A origin, Kan^R. Expresses *lacI*.	This study
p15A-lacI-PAR1	p15A origin, Kan^R, PAR1-based expression vector. Expresses *lacI*.	This study
pPAR1-S*acrAB*	p15A origin, Kan^R, expresses *Salmonella acrAB* from the PAR1 promoter. Expresses *lacI*.	This study
pET-26b(+)-PAR1-*pelB*^{SP}::anti-*IL-1β-6xhis* scFv	pBR322 origin, Kan^R, expresses scFv from the PAR1 promoter. Expresses *lacI*.	(16)
pET22b-PAR7-egfp	pBR322 origin, Amp^R, expresses eGFP from the PAR7 promoter. Expresses *lacI*^{q}.	(16)
pPAR7-E*acrAB*	pBR322 origin, Amp^R, expresses *E. coli acrAB* from the PAR7 promoter. Expresses *lacI*^{q}.	This study

**Oligonucleotides**		
**Name**	**Oligonucleotide sequence, 5' to 3'**	**Function**
pBAD30_Fwd_GA_1<br>pBAD30_Rev_GA_1	GATCAAAGGATCTTCCTGTCAGACCAAGTTTACTC<br>AACGTTGCGAAGCAAAGAGTTTGTAGAAACGCAAA	Amplification of pBAD30 backbone
pET26b_kan_Fwd_GA_1<br>pET26b_kan_Rev_GA_1	AACTTGGTCTGACAGGAAGATCCTTTGATCTTTTCTAC<br>ACTTCTGAGTTCGGCTTAGAAAAACTCATCGAGCATC	Amplification of Kan^R from pET26b
rrnB T1_Fwd<br>rrnB T1_Rev	GATGAGTTTTTCTAAGCCGAACTCAGAAGTGAAAC<br>GTTTCTACAAACTCTTTGCTTCGCAACGTTCAAAT	Amplification of *rrnB* T1 terminator from pBAD30
pBAD30-kanR_Fwd_GA<br>pBAD30-kanR_Rev_GA	CTCCCTTATGCGACTTTGGCGGATGAGAGAAGA<br>ACTCATTAGGCACCGGTAGCCGTCAAGTTGTCA	Amplification of pBAD30-kanR backbone
lacI_Fwd_GA_2<br>lacI_Rev_GA_2	TCTCTCATCCGCCAAAGTCGCATAAGGGAGAGC<br>CAACTTGACGGCTACCGGTGCCTAATGAGTGAGC	Amplification of lacI from pET26b
PAR1_Fwd_GA<br>PAR1_Rev_GA	CTCCCTTATGCGACTTGATGTCGGCGATATAGG<br>TCTCTCATCCGCCAAATGTATATCTCCTTCTTAAAGTTAAACAAA	Amplification of PAR1 promoter
p15A_Fwd_GA	ACTCATTAGGCACCGGTAGCCGTCAAGTTGTCA	

p15A_Rev_GA	ATATCGCCGACATCAAGTCGCATAAGGGAGAGC	Amplification of p15A-lacI backbone
p15A-PAR1_Fwd_GA<br>p15A-PAR1_Rev_GA	ACAGAACATCGCTGATTGGCGGATGAGAGAAGA<br>TCTGTTTTTGTTCATATGTATATCTCCTTCTTAAAGTTAAACA	Amplification of p15A-lacI-PAR1 backbone
acrA_Fwd_GA<br>acrB_Rev_GA	GAAGGAGATATACATATGAACAAAAACAGAGGGTTAAC<br>TCTCTCATCCGCCAATCAGCGATGTTCTGTCGA	Amplification of Salmonella *acrAB*
EacrAB-F<br>EacrAB-R	CCGGCTCGTATAATGTGTGGAAATTGTGAGCGGATAACAATTTCACACAGCTCGAGGTTTACATATGAAC<br>CCCGTTTAGAGGCCCCAAGGGGTTATGCTAGTTATTGCTCAGCGGTGCGTTGTATCAATGATGATCGAC	Amplification of *E. coli acrAB*
PAR7-F<br>PAR7-R	GAATGAAGATATCGAGCACAGCCATACTGTCGATCATCATTGATACAACGCACCGCTGAGCAAT<br>ACCGCCAGAGGCGTAAACCCTCTGTTTTTGTTCATATGTAAACCTCGAGCTGTGTGAAATTGTTATCCG	Amplification of PAR7 backbone

Supplemental Figure S1. Plasmid-encoded *acrAB* results in functional AcrAB-TolC which limits ethidium bromide accumulation.

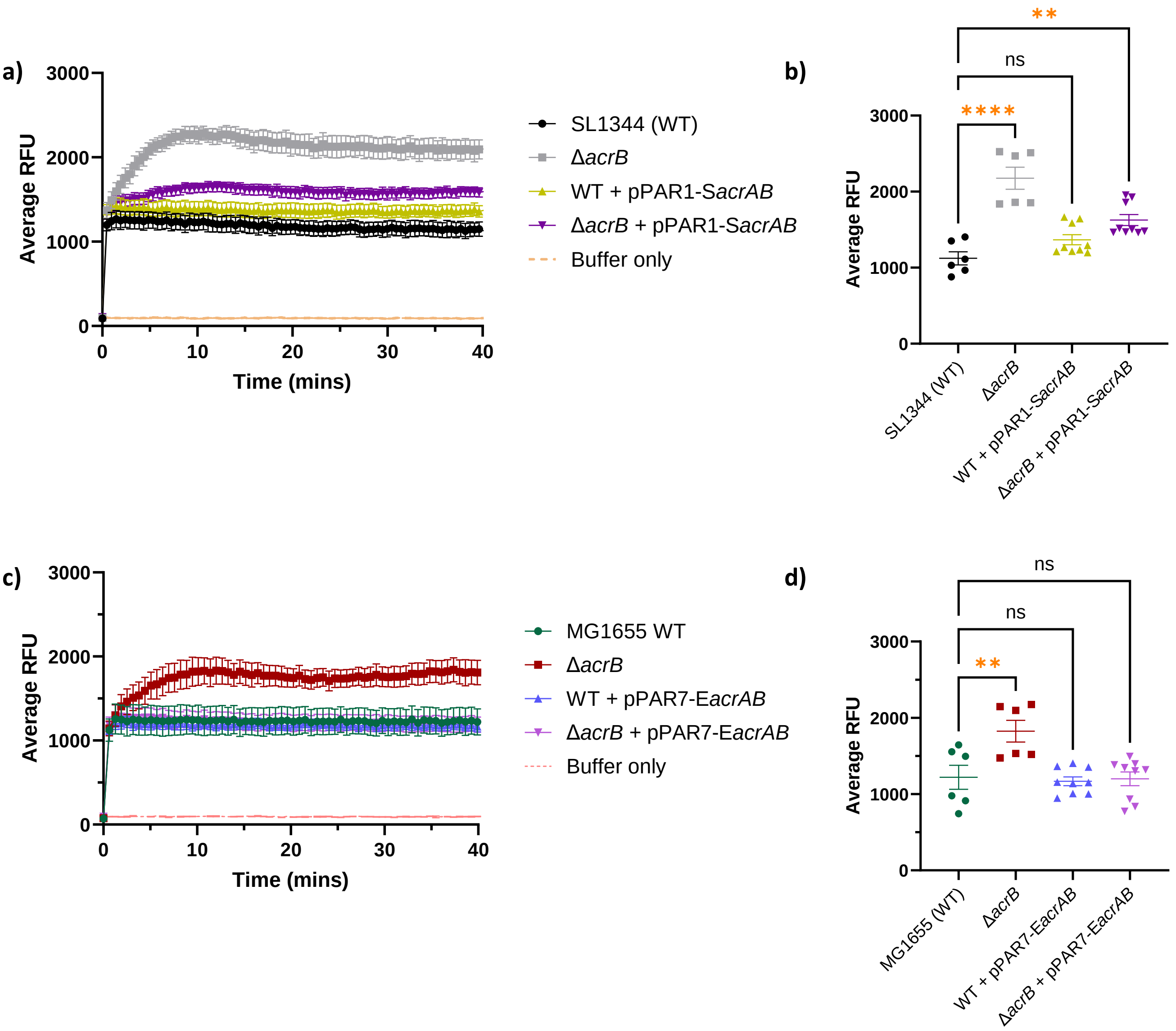

Accumulation of the dye and AcrAB-TolC efflux substrate ethidium bromide in (a,b) *Salmonella* and (c,d) *E. coli* wild type and Δ*acrB* strains in the absence or presence of plasmids expressing *acrAB* was measured over time (a,c). End point fluorescence of ethidium bromide is compared for each strain (b,d). For the timecourse data (a,c), mean fluorescence values ± SEM is plotted for n = 3. For the end point data (b.d), bars and whiskers represent mean ± SEM. Significance is tested using a one-way ANOVA: ns – not significant; **p<0.005; ****p<0.00005.

Supplemental Figure S2. Determination of membrane potential in *E. coli*.

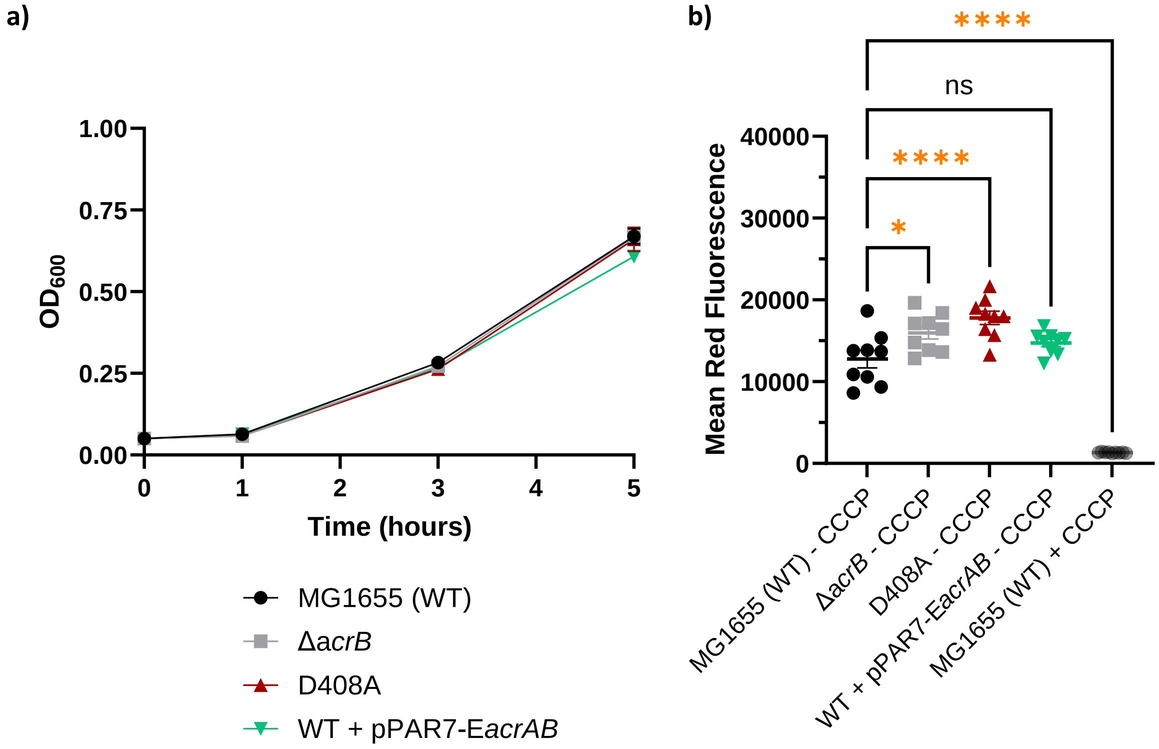

E. coli MG1655 (wild type), Δ*acrB*, *acrB* D408A, and MG1655 transformed with pPAR7-E*acrAB* (expressing *E. coli acrAB*) cells were grown in MOPS minimal medium; (a) growth was monitored by measuring OD_{600}. Samples were taken after 5 hours growth and membrane potential was determined using flow cytometry and $DiSC_3(5)$ (b). The red fluorescence, corresponding to membrane potential, is shown for each strain. The protonophore CCCP was used as a control to collapse membrane potential. Significance was tested using a one-way ANOVA; * $p \leq 0.05$; **** $p < 0.00005$.

References

1. Smith C, Stringer AM, Mao C, Palumbo MJ, Wade JT. 2016. Mapping the regulatory network for *Salmonella enterica* serovar typhimurium invasion. MBio 7:e01024-16.

2. Gunn JS. 2008. The *Salmonella* PmrAB regulon: lipopolysaccharide modifications, antimicrobial peptide resistance and more. Trends Microbiol 16:284–290.

3. Cohen H, Adani B, Cohen E, Piscon B, Azriel S, Desai P, Bähre H, McClelland M, Rahav G, Gal-Mor O. 2022. The ancestral stringent response potentiator, DksA has been adapted throughout *Salmonella* evolution to orchestrate the expression of metabolic, motility, and virulence pathways. Gut Microbes 14:1997294.

4. Kim MJ, Lim S, Ryu S. 2008. Molecular analysis of the *Salmonella typhimurium tdc* operon regulation. J Microbiol Biotechnol 18:1024–1032.

5. Fink RC, Evans MR, Porwollik S, Vazquez-Torres A, Jones-Carson J, Troxell B, Libby SJ, McClelland M, Hassan HM. 2007. FNR is a global regulator of virulence and anaerobic metabolism in *Salmonella enterica* serovar typhimurium (ATCC 14028s). J Bacteriol 189:2262–2273.

6. Frye J, Karlinsey JE, Felise HR, Marzolf B, Dowidar N, McClelland M, Hughes KT. 2006. Identification of new flagellar genes of *Salmonella enterica* serovar typhimurium. J Bacteriol 188:2233–2243.

7. Karavolos MH, Spencer H, Bulmer DM, Thompson A, Winzer K, Williams P, Hinton JCD, Khan CMA. 2008. Adrenaline modulates the global transcriptional profile of *Salmonella* revealing a role in the antimicrobial peptide and oxidative stress resistance responses. BMC Genomics 9:458.

8. Kröger C, Colgan A, Srikumar S, Händler K, Sivasankaran SK, Hammarlöf DL, Canals R, Grissom JE, Conway T, Hokamp K, Hinton JCD. 2013. An infection-relevant transcriptomic compendium for *Salmonella enterica* serovar Typhimurium. Cell Host Microbe 14:683–695.

9. Liu R, Blackwell TW, States DJ. 2001. Conformational model for binding site recognition by the *E. coli* MetJ transcription factor. Bioinformatics 17:622–633.

10. McDermott JE, Yoon H, Nakayasu ES, Metz TO, Hyduke DR, Kidwai AS, Palsson BO, Adkins JN, Heffron F. 2011. Technologies and approaches to elucidate and model the virulence program of *Salmonella*. Front Microbiol 2:121.

11. Mukhopadhyay S, Schellhorn HE. 1994. Induction of *Escherichia coli* hydroperoxidase I by acetate and other weak acids. J Bacteriol 176:2300–2307.

12. Weber H, Polen T, Heuveling J, Wendisch VF, Hengge R. 2005. Genome-wide analysis of the general stress response network in *Escherichia coli*: σ^S-dependent genes, promoters, and sigma factor selectivity. J Bacteriol 187:1591–1603.

13. Klein G, Kobylak N, Lindner B, Stupak A, Raina S. 2014. Assembly of lipopolysaccharide in *Escherichia coli* requires the essential LapB heat shock protein. J Biol Chem 289:14829–14853.

14. Fàbrega A, Vila J. 2013. *Salmonella enterica* serovar Typhimurium skills to succeed in the host: Virulence and regulation. Clin Microbiol Rev 26:308–341.

15. Guzman LM, Belin D, Carson MJ, Beckwith J. 1995. Tight regulation, modulation, and high-level expression by vectors containing the arabinose P(BAD) promoter. J Bacteriol 177:4121–4130.

16. Hothersall J, Godfrey RE, Fanitsios C, Overton TW, Busby SJW, Browning DF. 2021. The PAR promoter expression system: Modified *lac* promoters for controlled recombinant protein production in *Escherichia coli*. N Biotechnol 64:1–8.
